# MaxEnt model-based prediction of potential suitable habitats of three Trichosanthes L. species in China under future climate change scenarios

**DOI:** 10.3389/fpls.2026.1748030

**Published:** 2026-02-06

**Authors:** Xiaomeng Wang, Yuanyuan Ding, Chunfeng Duan, Yunchuan Xu, Chen Zhang, Zihan Wang

**Affiliations:** 1 Reading College, Nanjing University of Information Science and Technology, Nanjing, China; 2 School of Geographical Sciences, Nanjing University of Information Science and Technology, Nanjing, China; 3 Anhui Key Laboratory of Atmospheric Science and Satellite Remote Sensing, Anhui Institute of Meteorological Sciences, Hefei, China; 4 Taizhou Yuhuan Meteorological Bureau of Zhejiang, Taizhou, China; 5 Jiangsu Provincial University Key Laboratory of Agricultural and Ecological Meteorology, Nanjing University of Information Science and Technology, Nanjing, China

**Keywords:** climate change, MaxEnt, prediction, suitable habitat, Trichosanthes

## Abstract

**Introduction:**

Global warming is reshaping species’ distributions, threatening the habitats of three medicinal lianas in the genus *Trichosanthes*, and highlighting the need to predict their potential suitable areas under future climate conditions. This study focuses on *Trichosanthes rubriflos, Trichosanthes rosthornii, and Trichosanthes kirilowii (T. rubriflos, T. rosthornii, and T. kirilowii)*, aiming to understand how climate change will affect their distributions and which climatic drivers primarily influence their habitat suitability.

**Methods:**

Present and future suitability patterns were delineated using an optimized MaxEnt model, driven by contemporary climate data and four Shared Socioeconomic Pathway (SSP) scenarios (SSP1-2.6, SSP2-4.5, SSP3-7.0, SSP5-8.5). Model performance was enhanced through parameter tuning and evaluation, and the principal climatic drivers of habitat suitability were identified from the fitted models.

**Results:**

The tuned MaxEnt models reliably predicted habitat suitability. *T. rubriflos* showed largely contiguous suitability across the low-mountain and hilly belts of South and Southwest China. *T. rosthornii* was concentrated along the eastern Loess Plateau and the mountains of North China, while *T. kirilowii* exhibited a patchy distribution across the middle-lower Yangtze region. Temperature seasonality emerged as the primary driver for *T. rubriflos*, while annual precipitation was the most influential factor for *T. rosthornii* and *T. kirilowii*. Across all scenarios, habitat expansions generally outpaced contractions, with species shifting poleward and upslope. Scenario-specific responses included the expansion of *T. rubriflos* in South China and the northward movement of *T. rosthornii* under SSP3-7.0, as well as the expansion of *T. kirilowii* into southwestern mountain systems, coupled with partial contraction on the North China Plain under SSP5-8.5.

**Discussion:**

Distributions of the three *Trichosanthes* species are chiefly shaped by temperature seasonality (TS) and annual precipitation (AP), with species-specific sensitivities: TS dominates *T. rubriflos*, AP (plus cold stress) constrains *T. rosthornii*, and *T. kirilowii* shows mid-range (double-threshold) responses. Across 2061–2080, ranges generally expand and shift poleward and upslope; suitability peaks under SSP3-7.0 for *T. rubriflos* and *T. rosthornii*, and under SSP5-8.5 for *T. kirilowii*.These findings provide a scientific basis for identifying future conservation priorities, guiding *in situ* protection in persistent or newly suitable regions, and informing climate-adaptive management of medicinal *Trichosanthes* species.

## 1 Introduction

Rising greenhouse gas emissions are driving a sustained increase in global mean temperature (IPCC, 2021). This warming is reshaping species’ geographic distributions (Randin et al., 2020; Vitasse et al., 2021) and has prompted widespread shifts towards higher latitudes and elevations (He et al., 2019). For the three Asian endemic medicinal lianas *Trichosanthes rubriflos, Trichosanthes rosthornii, and Trichosanthes kirilowii (T. rubriflos, T. rosthornii, and T. kirilowii)*, niches are closely associated with forest gaps and edge habitats in tropical and subtropical forests (Ingwell et al., 2010), where these climbers maintain intimate, both symbiotic and competitive, relationships with trees. Climate-induced thermal variability and extreme events disrupt this balance and directly threaten climbing habitats. In addition, limited dispersal capacity constrains timely adaptation. Given their pharmacological importance and growing market demand, assessing their potential suitable habitats in China under future climate scenarios is therefore essential.

Species distribution models (SDMs) relate occurrence records to environmental predictors to infer ecological niches and estimate potential distributions under present and future climates, and they now serve as standard tools for assessing ecological impacts of climate change (Elith et al., 2011; Hampe, 2004; Zhang et al., 2020).Common approaches include BIOCLIM, boosted regression trees (BRT), CLIMEX, ecological niche factor analysis (ENFA), generalized linear models (GLM), GARP, MaxEnt, and random forests (RF). SDM research originated in studies of plant-environment gradients, initially emphasizing species’ response curves (Whittaker, 1956; MacArthur, 1958). Advances in computing and statistics in the 1980s shifted SDMs toward prediction, and the rapid growth of GIS in the 1990s spurred the proliferation of environment-driven SDMs and associated software (Li et al., 2013; Xu et al., 2015). SDMs are now widely used to forecast distributional responses to climate change (Park et al., 2022; Shi et al., 2024), including paleo-modern range coupling (Svenning et al., 2011), potential suitability mapping, spatial distribution and quality assessment of medicinal plants (Zhao et al., 2017), invasion risk and habitat suitability of alien species (Xian et al., 2022) and multi-model pattern analyses (Coro et al., 2024). Among these methods, MaxEnt offers distinct advantages for presence-only and small-sample data, accommodates both continuous and categorical predictors, and captures complex species-environment relationships; built on the maximum-entropy principle, it delivers stable performance with high predictive accuracy (Phillips et al., 2006; Elith et al., 2011; Merow et al., 2013).

An increasing number of recent studies use MaxEnt to evaluate future suitability for herbaceous medicinal plants—for example, projected high-elevation contraction of *Panax notoginseng*, shrinkage in *Glycyrrhiza* spp., and northward expansion of *Angelica sinensis* (Guo et al., 2024; Ma et al., 2025; Xi et al., 2025). By contrast, work on medicinal lianas remains fragmented, often confined to single species or local systems, and lacks quantitative, multi-species analyses of community-level suitability and distributional responses across scenarios. Integrating multi-species data with niche models to predict liana community suitability under different climate futures would clarify habitat-adaptation mechanisms and provide an evidence base for coordinated conservation and applied practices, including urban greening.

As cucurbitaceous medicinal lianas, *T. rubriflos*, *T. rosthornii*, and *T. kirilowii* possess notable pharmacological activities (e.g., cardioprotective and antitumor effects) and are widely cultivated, thereby conferring considerable economic and medicinal value. Ecologically, these climbers engage in distinctive interspecific interactions with trees; by competing for light and space, their climbing habit shapes complex competitive-symbiotic dynamics that influence forest structure and functioning. However, most liana research has focused on their impacts on forest dynamics (Laurance et al., 2014; Schnitzer et al., 2014a) and, in modelling practice, often treats lianas as a single functional group (Porcia e Brugnera et al., 2019). Indeed, nearly fifty years ago it was noted that “liana ecology remains largely unknown” (Jacobs, 1976; Schnitzer et al., 2014b). In addition to their medicinal value, *Trichosanthes* species also play important ecological roles in natural ecosystems. As climbing plants, they contribute to forest vertical structure and influence light availability and plant–plant interactions. Their flowers and fruits may also provide resources for insects and other small animals, supporting local food webs. Therefore, changes in their distribution may have broader implications for ecosystem structure and stability. A deeper understanding of Trichosanthes, encompassing its ecological, economic, and medicinal value, will refine liana research frameworks, shed light on forest ecosystem functioning, and promote sustainable use and conservation.

Although MaxEnt has been widely used to predict the distribution of single liana species, integrated multi-species analyses remain scarce, and the habitat preferences and key environmental drivers of three *Trichosanthes* species have not been systematically clarified. Extensive occurrence records for *Trichosanthes rubriflos* (*T. rubriflos*), *T. rosthornii*, and *T. kirilowii* were compiled and combined with 31 environmental variables to build MaxEnt-based habitat suitability models to predict their current and future suitable areas. Simulations cover 2060–2080 under four CMIP6 Shared Socioeconomic Pathways (SSPs): SSP1-2.6 (SSP126), SSP2-4.5 (SSP245), SSP3-7.0 (SSP370), and SSP5-8.5 (SSP585). This study not only reveals the spatiotemporal dynamics of the distributions of the three *Trichosanthes* species under climate change but also provides an evidence base for coordinated multi-species conservation.Therefore, this study aims to address the following scientific questions: (1) how the habitat suitability patterns of the three *Trichosanthes* species differ under current climatic conditions; (2) which key environmental variables primarily drive their distributions; and (3) how their suitable habitats are expected to shift under different future SSP scenarios.

## 2 Materials and methods

### 2.1 Study area

China spans a vast territory (3°51′-53°33′ N, 73°40′-135°02′ E) with marked environmental heterogeneity. The three *Trichosanthes* species investigated here occur primarily in southern China, especially South China. This region is a transitional zone between tropical and subtropical monsoon climate, with mean annual temperature of 16.5-24.8 °C and annual precipitation of 1, 100-2, 800 mm. Topography grades from the western plateau to the south-eastern coastal plains, producing distinct temperature-moisture gradients and diverse ecological settings. These conditions provide the context for assessing potential shifts in suitable habitats of the three species under future climate scenarios.

### 2.2 Study framework

**Figure 1:**
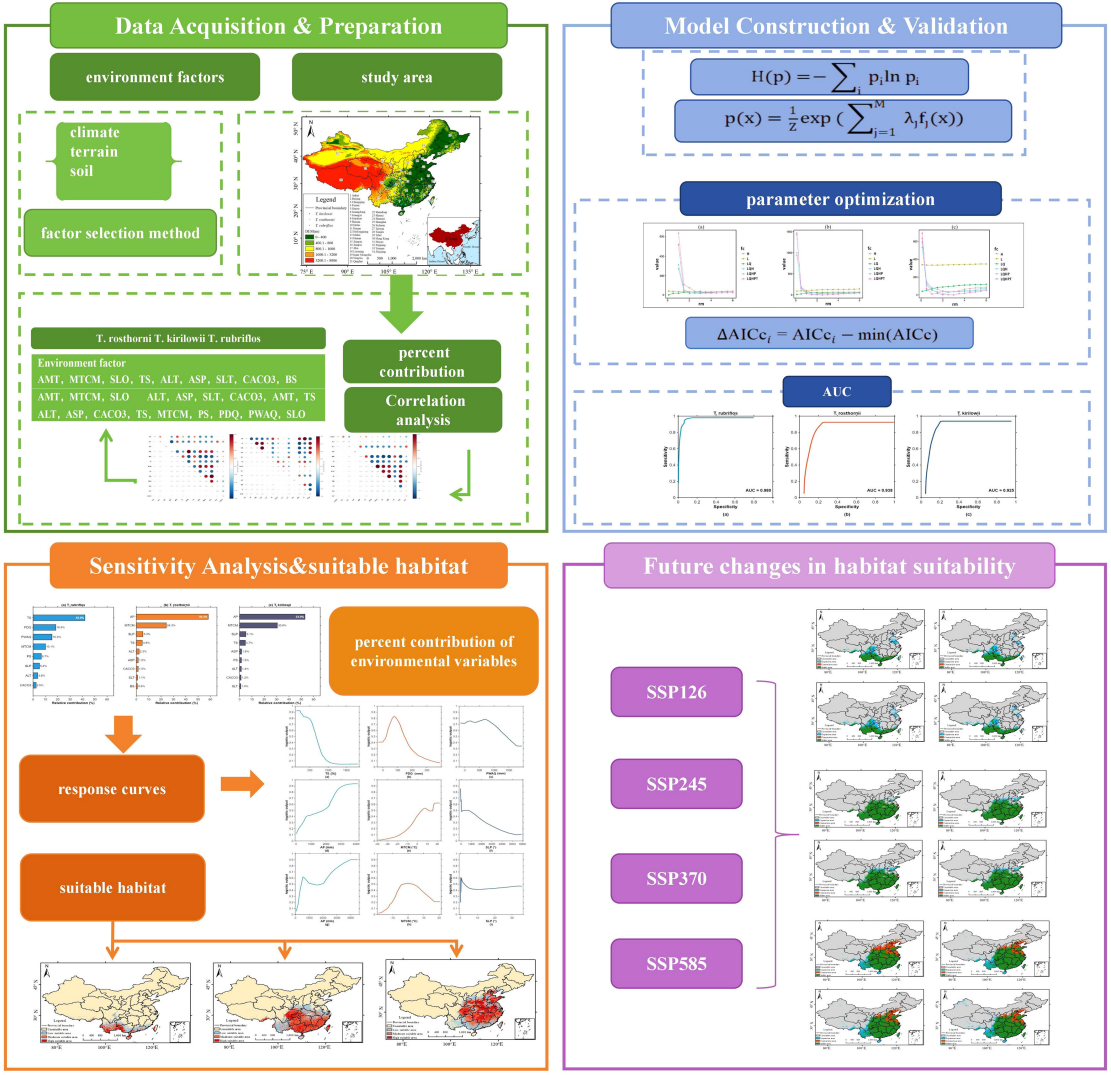
Workflow of the research framework, including occurrence data collection, environmental variable screening, MaxEnt model optimization and validation, and projections of current and future habitat suitability under different SSP scenarios.

The study proceeded in four stages (**Figure 1**). (1) Data acquisition & preparation. We compiled georeferenced occurrences of *Trichosanthes* and assembled environmental predictors covering climate, terrain, and soil. Collinearity was screened via correlation analysis, and retained variables were standardized for modelling. (2) Model construction & validation. Species distributions were modeled with MaxEnt; key settings were tuned by response diagnostics and ΔAICc minimization, and predictive performance was evaluated using AUC and threshold-dependent metrics. (3) Sensitivity analysis & current suitability. We quantified percent contributions and permutation importance, interpreted response curves, and mapped present-day suitability for each species. (4) Future prediction. Using downscaled WorldClim projections, we projected habitat suitability under SSP1-2.6, SSP2-4.5, SSP3-7.0, and SSP5-8.5, and compared spatial shifts to assess potential expansions, contractions, and hotspots of change.

### 2.3 Data

#### 2.3.1 Occurrence data collection

**Figure 2:**
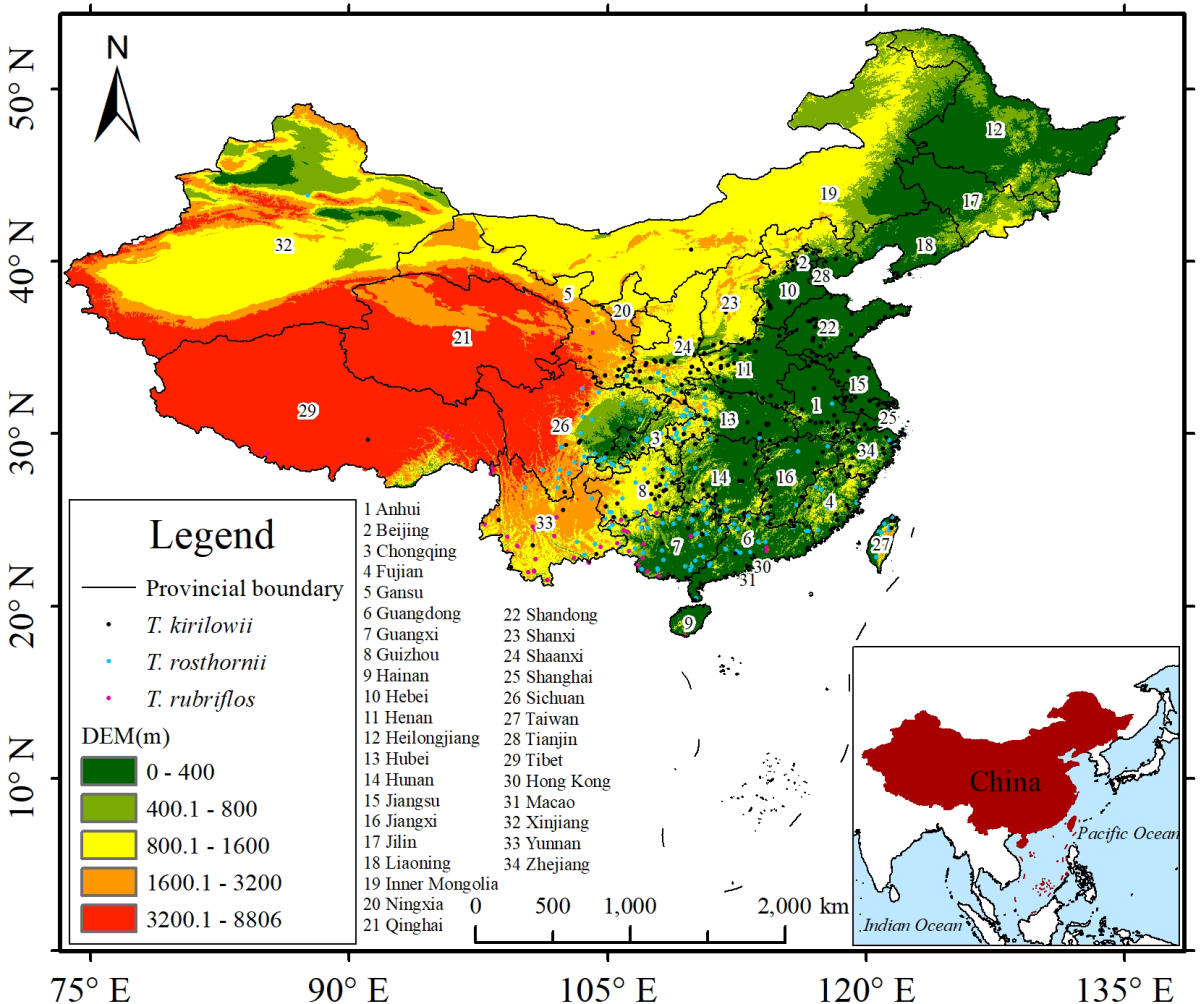
Spatial distribution of occurrence records of the three *Trichosanthes* species in China used for MaxEnt modeling.

Georeferenced occurrence records were compiled from multiple reliable sources, including historical surveys of medicinal plant resources in China, germplasm resource inventories, and peer-reviewed literature on the three focal *Trichosanthes* species. In addition, specimen databases were queried through the Global Biodiversity Information Facility (GBIF, https://www.gbif.org), the Chinese Virtual Herbarium (CVH, https://www.cvh.ac.cn), the Educational Specimen Resource Center at Sichuan University (https://mnh.scu.edu.cn), and the China Nature Reserve Resources Platform (PAPC, http://www.papc.cn) to obtain collection localities. After data acquisition, occurrence records were filtered and preprocessed by removing entries with insufficient coordinate precision, obviously erroneous coordinates, or spatially-duplicated records.The final dataset included 59 records of *T. rubriflos*, 261 records of *T. rosthornii* and 361 records of *T. kirilowii*, (**Figure 2**). These procedures ensured the reliability of the occurrence data and minimized the influence of erroneous and duplicate records.

#### 2.3.2 Collection and preprocessing of environmental variables

Current climatic variables (1970-2000) were obtained from the WorldClim v2.1 database (Fick and Hijmans, 2017). Future climatic variables (2061-2080) were taken from downscaled CMIP6 projections in WorldClim v2.1 for the model BCC-CSM2-MR under four Shared Socioeconomic Pathways (SSPs): SSP1-2.6, SSP2-4.5, SSP3-7.0, and SSP5-8.5.The resolution of these climate layers (2.5 arc-minutes) is appropriate for species distribution modeling.The differences among these SSP scenarios, including variations in projected temperature and precipitation patterns, were explicitly incorporated into predictions of future habitat suitability for the three species.Previous evaluations indicate that BCC-CSM2-MR performs well in simulating the East Asian summer monsoon and associated precipitation and exhibits high predictive skill for regional climate over China (Sang et al., 2021; Guo et al., 2021).To reduce multicollinearity and avoid overfitting, candidate predictors were screened using Pearson correlation analysis, and highly correlated variables (|r| > 0.8) were removed. Variable importance was further assessed based on their contributions in preliminary MaxEnt models, and variables with negligible influence were excluded. The final set of predictors therefore balances ecological relevance and statistical robustness. All environmental predictors used in the models are summarized in **Table 1**.

**Table 1:** Environmental variables for the three *Trichosanthes* species.

Type	Abbreviation	Factors	Data sources	Year	Unit
Climaticvariables	AMT	Annual mean temperature	World Clim: Global Climate Data Version2.0 http://www.worldclim.org/(accessed:2024-05-24)	1970-2000	°C
	MDR	Mean diurnal range			°C
	ISO	Isothermality = (MDR/TAR) ×100			%
	TS	Temperature seasonality (standard Deviation100)			%
	MTWM	Max temperature of warmest month			°C
	MTCM	Min temperature of coldest month			°C
	TAR	Temperature annual range=MTWM-MTCM			°C
	MTWQ	Mean temperature of wettest quarter			°C
	MTDQ	Mean temperature of driest quarter			°C
	MTWAQ	Mean temperature of warmest quarter			°C
	MTCQ	Mean temperature of coldest quarter			°C
	AP	Annual precipitation			mm
	PWM	Precipitation of wettest month			mm
	PDM	Precipitation of driest month			mm
	PS	Precipitation seasonality (coefficient of variation)			%
	PWQ	Precipitation of wettest quarter			mm
	PDQ	Precipitation of driest quarter			mm
	PWAQ	Precipitation of warmest quarter			mm
	PCQ	Precipitation of coldest quarter			mm
Terrain variables	SLP	Slope	World Clim: Global Climate Data Version2.0, Web: http://www.worldclim.org/(accessedon24May2024)	2010	°
	ALT	Altitude			m
	ASP	Aspect			
Soil variables	PH	PH value	HWSD: Harmonized World Soil DatabaseVersion2.0, Web: http://www.fao.org/soils-portal/soil-survey/soil-mAPS-and-databases/harmonized-world-soil-database-v12/en/	2012	—
	OC	Organic content			%
	CACO3	Calcium carbonated content			%
	BS	Base saturation			%
	ESP	Exchangeable sodium percentage			%
	SLT	Silt			%
	TEB	Total exchangeable bases			cmolc kg^-^¹
	UST	Usda soil taxonomy			%

### 2.4 Methods

#### 2.4.1 Principle of the MaxEnt model

This study used MaxEnt v3.4.4, based on the maximum-entropy principle, to predict the potential distribution of *Trichosanthes* species. Its core idea is to maximize information entropy subject to constraints that the expected values of environmental features, as estimated from species-occurrence samples, equal those under the model (Equation 1).

(1) $$H(p)=-\sum_{i}p_{i}\ln p_{i}$$

The resulting “most uniform” (least assumptive) probability distribution takes the form as follows (Equation 2):

(2) $$p(x)=\frac{1}{Z}\;\exp!(\sum_{j=1}^{M}\lambda_{j}f_{j}(x))$$

where $f_{j}(x)$ denotes the jth environmental feature function, $\lambda_{j}$ is the corresponding Lagrange multiplier, and Z is the normalizing constant. This approach avoids making unwarranted inferences when data are limited and reduces overfitting risk. MaxEnt also integrates a jackknife procedure to compute the independent contribution of each predictor, enabling variable-importance ranking, and supports multiple feature classes (linear, quadratic, hinge, product, and threshold) for model optimization.

#### 2.4.2 Model accuracy evaluation

Model performance was evaluated primarily using the area under the receiver operating characteristic (ROC) curve (AUC) and its standard deviation (SD). An AUC closer to 1 indicates stronger discrimination, and higher values reflect greater reliability and predictive skill. Using commonly adopted thresholds, AUC < 0.6 indicates poor performance; 0.6 ≤ AUC < 0.8 suggests moderate reliability; 0.8 ≤ AUC < 0.9 denotes good performance; and AUC ≥ 0.9 indicates excellent discrimination (Xiao et al., 2019). To further optimize model settings, the small-sample-corrected Akaike information criterion (AICc) was calculated for competing specifications, and relative support was assessed using $\Delta AICc_{i}\;$and Akaike weights (Equation 3).

(3) $$\Delta AICc_{i}=AICc_{i}-min(AICc)$$

When  ΔAICc  < 2, alternative models are considered to have equivalent support (Symonds and Moussalli, 2011).

#### 2.4.3 Screening of environmental variables

**Figure 3:**
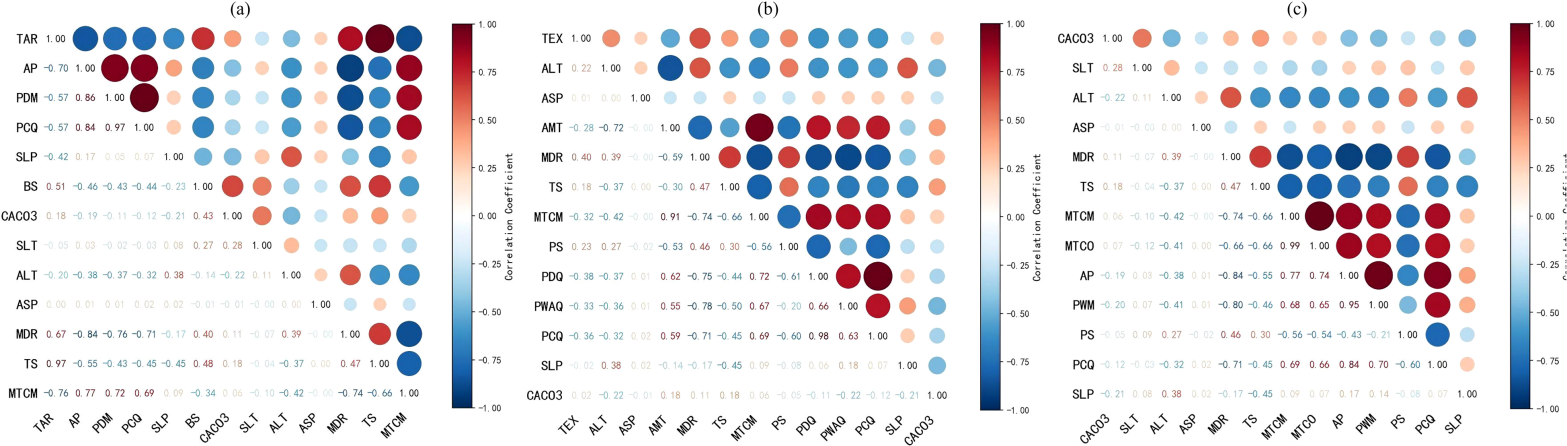
Pearson correlation heatmap of environmental variables used in the MaxEnt models, supporting variable selection and reduction of multicollinearity for the three Trichosanthes species: **(a)** *T. rubriflos*; **(b)** *T. rosthornii*; **(c)** *T. kirilowii*.

Occurrence records for the three *Trichosanthes* species were integrated with 31 climatic, soil and topographic predictors in MaxEnt using a a training (75%) and testing (25%) set (Zhou et al., 2022). Predictor importance was quantified with jackknife tests and model performance evaluated using ROC/AUC; predictors with zero contribution were removed iteratively. Multicollinearity was assessed via Pearson correlation coefficients, treating |r| ≥ 0.8 as high; within each correlated pair, the predictor with the greater MaxEnt contribution and greater ecological interpretability was retained, and the procedure repeated until all pairwise |r| < 0.8 (**Figure 3**). Nine environmental predictors were ultimately retained for each species (*T. rubriflos, T. rosthornii, T. kirilowii*) for subsequent modelling and climate projections (**Table 2**).

**Table 2:** Environmental variables retained after screening and used for model construction for the three *Trichosanthes* species.

Species	Environmental factor
*T.rosthorni*	AMT, MTCM, SLP, TS, ALT, ASP, SLT, CACO3, BS
*T.kirilowii*	AMT, MTCM, SLP, ALT, ASP, SLT, CACO3, AMT, TS
*T.rubriflos*	ALT, ASP, CACO3, TS, MTCM, PS, PDQ, PWAQ, SLP

#### 2.4.4 Model tuning and evaluation

Generally, the probability predictions of uncalibrated models may not align with the true distribution of species (Mendes et al., 2020). In this study, the R package ENMeval was used as a framework to explore and compare alternative MaxEnt parameter settings for feature classes (FC) and regularization multipliers (RM) (Chala et al., 2016; Merow et al., 2014; Muscarella et al., 2014). Previous studies have shown that RM values are often higher than the default of 1, with optimal performance frequently reported within the range of 2–4 (Moreno-Amat et al., 2015). To allow sufficient flexibility in model complexity, RM values were therefore explored over a broader range.

Six FC combinations—linear (L), linear–quadratic (LQ), hinge (H), LQH, LQHP, and LQHPT—were evaluated in combination with RM values ranging from 0.1 to 6.0 at 0.5 intervals, resulting in 78 candidate parameter configurations. All models were assessed using test-set AUC, and the parameter combination yielding the highest predictive performance was selected for final model construction.

## 3 Results and analysis

### 3.1 Model tuning and evaluation

**Figure 4:**
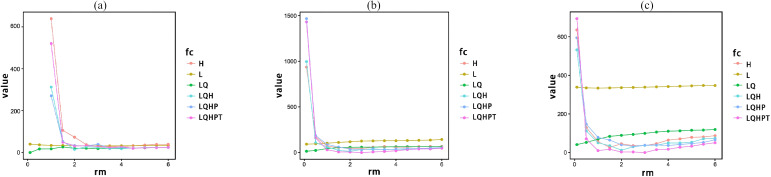
MaxEnt parameter optimization results for the three *Trichosanthes* species—grid search over the regularization multiplier (RM) and feature-class sets (FC), with the optimal RM-FC combinations highlighted for: **(a)** *T. rubriflos*; **(b)** *T. rosthornii*; **(c)** *T. kirilowii*.

**Figure 5:**
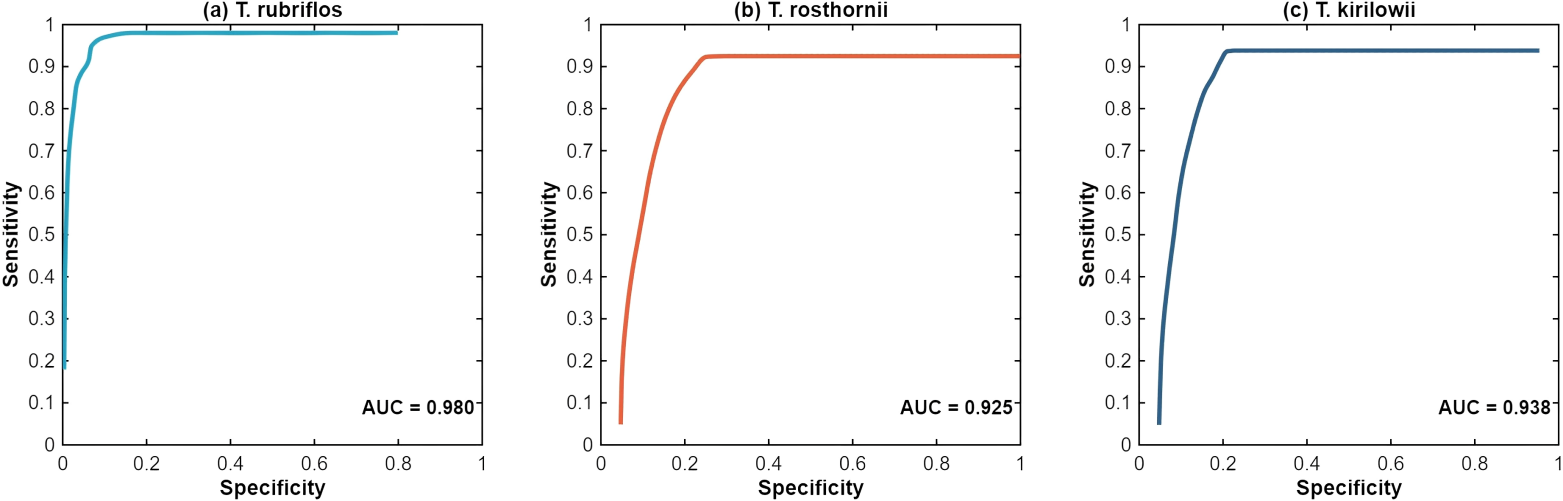
Mean receiver operating characteristic (ROC) curves and area under the curve (AUC) for the three *Trichosanthes* species: **(a)** *T. rubriflos*; **(b)** *T. rosthornii*; **(c)** *T. kirilowii*. The blue curve denotes the mean ROC for each model.

To enhance model reliability, a grid search was performed over the MaxEnt regularization multiplier (RM; 0.1-6.0 in 0.5 increments; 13 levels) and feature classes (FCs: L, LQ, H, LQH, LQHP, LQHPT), yielding 78 parameter configurations (**Figure 4**). Under the optimal parameterization, the test-set AUCs for *T. rubriflos, T. rosthornii* and *T. kirilowii* were 0.980, 0.938 and 0.925, respectively (**Figure 5**), indicating excellent discrimination and robust performance of the tuned models.

### 3.2 Present-day suitability patterns of the three *Trichosanthes* species

**Figure 6:**
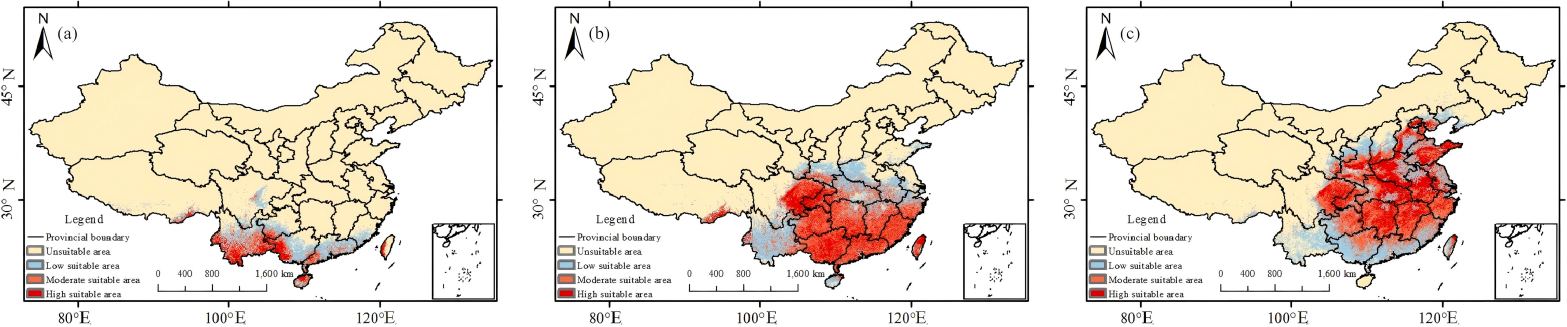
Present-day habitat suitability maps for the three *Trichosanthes* species: **(a)** *T. rubriflos*; **(b)** *T. rosthornii;* **(c)** *T. kirilowii*.

Using MaxEnt outputs, the areas and spatial patterns of high-, medium-, and low-suitability zones for *T.*rubriflos, *T. rosthornii*, and *T. kirilowii* under the present climate are compared (**Figure 6**), with suitability classes determined using the natural breaks (Jenks) method, which objectively sets class boundaries by minimizing within-class variance and maximizing between-class differences, providing a basis for subsequent species-specific suitability analyses.

For *T. rubriflos*, the high-suitability area reaches 581.765 × 10² km², centered on the Yunnan-Guizhou Plateau and parts of Hainan. Although unsuitable areas account for 76.3% of the study area, high-suitability zones show a patchy pattern, including a continuous belt in Yunnan; the combined medium and low-suitability area (145.460 × 10² km²) form a rim around the core. *T. rosthornii* has 103.037 × 10² km² (10.7%) of high suitability, mainly in Guizhou, Guangxi and southern Guangdong; the medium-suitability zone (103.803 × 10² km²) extends westwards to Yunnan and northwards to Hubei-Henan-Anhui, whereas the low-suitability zone (67.717 × 10² km²) is scattered across Henan, Shaanxi and Sichuan. For *T. kirilowii*, the high-suitability area is 44.013 × 10² km² (4.6%), with combined medium and low suitability totaling 32.842 × 10² km²; its high-suitability belt across the hilly-plain zone of the middle-lower Yangtze grades into a mosaic, fragmented pattern along the northern and south-western margins.

Overall, *T. rubriflos* has a core suitability hotspot centered on the Yunnan-Guizhou Plateau, while *T. rosthornii* and *T. kirilowii* exhibit the broadest ranges among the three and closely follow the humid subtropical climate zone.

### 3.3 Contributions of environmental predictors

**Figure 7:**
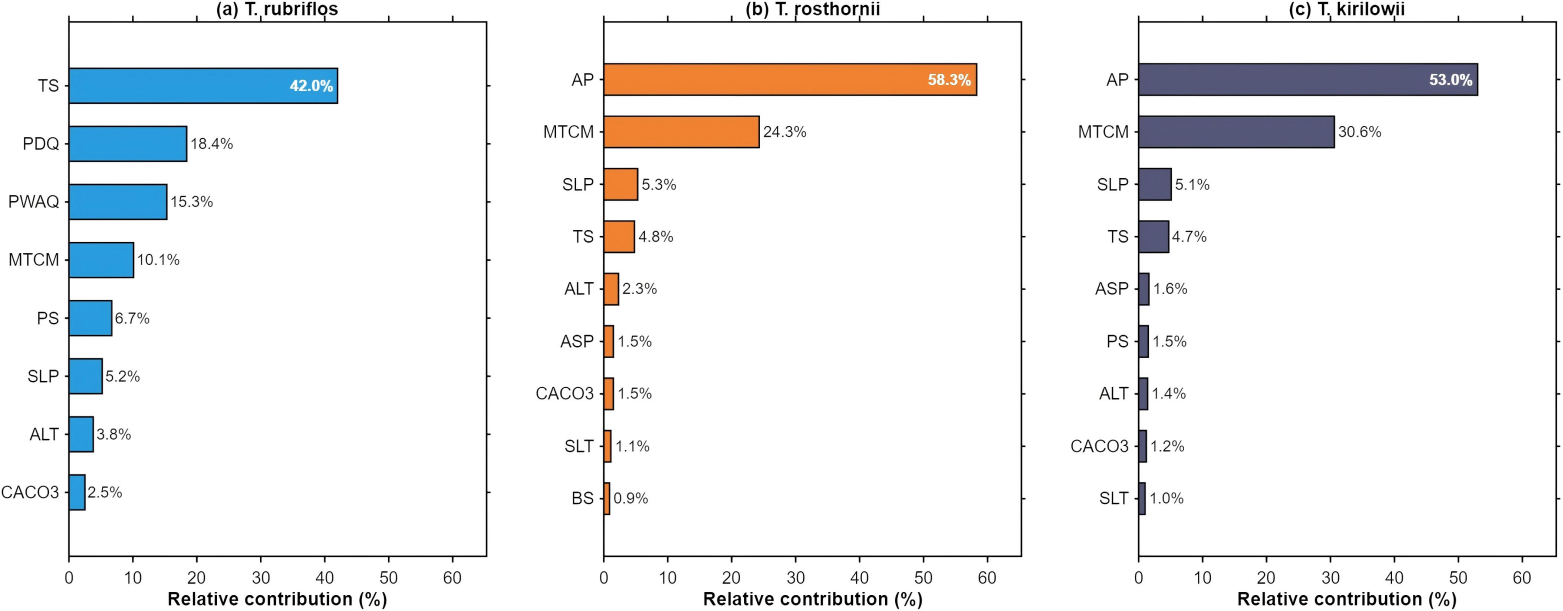
Relative contributions of key environmental predictors to the potential distributions of the three *Trichosanthes* species: **(a)** *T. rubriflos*; **(b)** *T. rosthornii*; **(c)** *T. kirilowii*.

**Figure 8:**
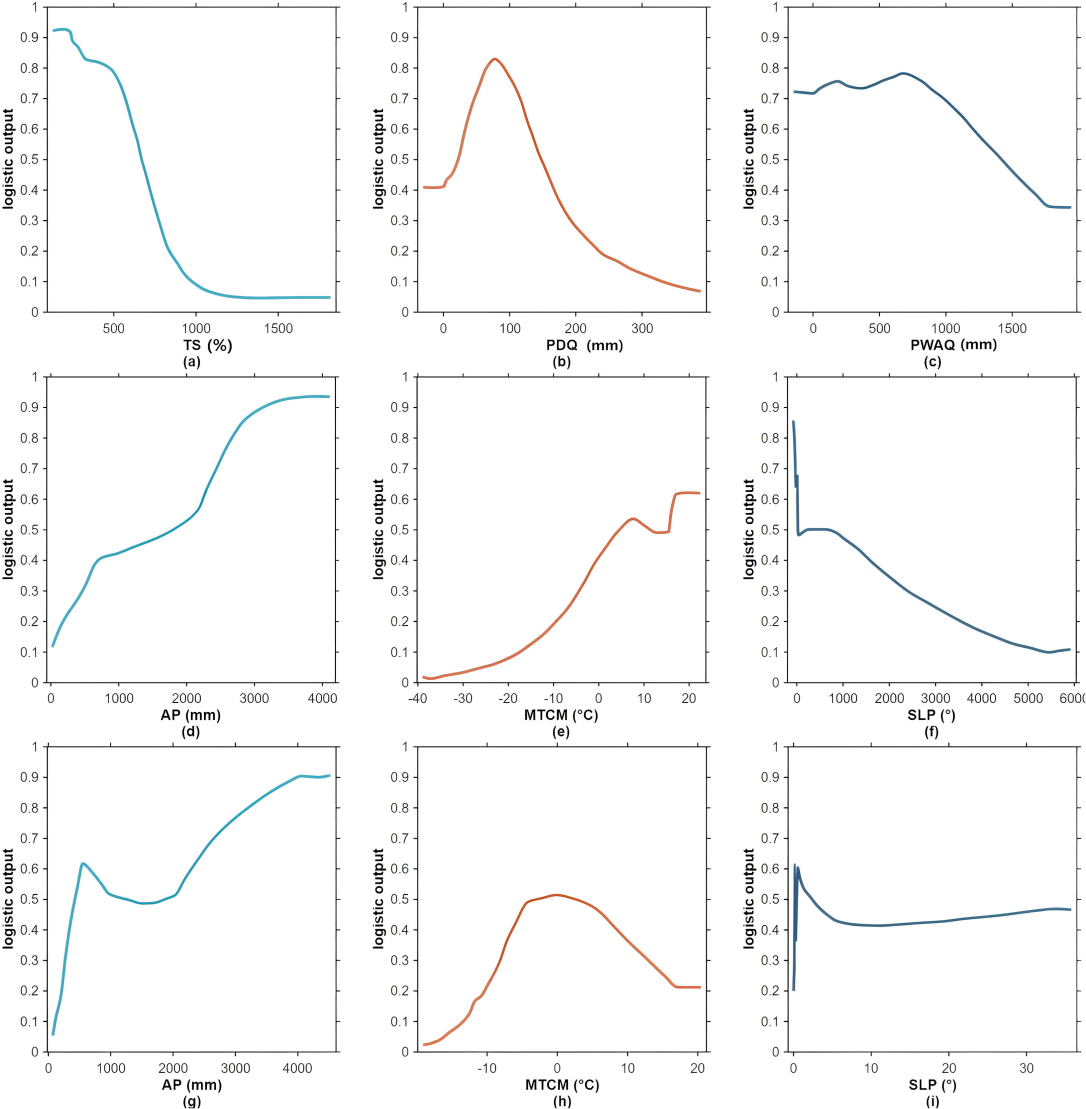
Response curves of the most influential environmental variables for the three *Trichosanthes* species: **(a-c)** *T. rubriflos*; **(d-f)** *T. rosthornii*; **(g-i)** *T. kirilowii*.

The potential distributions of the three *Trichosanthes* species are shaped by multiple environmental factors (**Figure 7**). For *T. rubriflos*, habitat suitability is primarily driven by temperature seasonality (TS, 42.0%) and precipitation of the driest quarter (PDQ, 18.4%), which jointly account for 60.4% of the total contribution, with precipitation of the warmest quarter (PWQ, 15.6%) providing additional explanatory power. Response curves for key predictors (**Figures 8a–c**) indicate a high-suitability range at TS of 125-630 (units: SD × 100, i.e., the standard deviation of monthly mean temperature multiplied by 100) and PDQ of 40–150 mm; within these ranges, predicted suitability (logistic output) remains high. This pattern is consistent with findings for *Actinidia eriantha* and related subtropical taxa, underscoring the pervasive role of TS and seasonal precipitation in shaping distributions in the subtropics (Guo et al., 2022).

For *T. rosthornii*, suitability is primarily driven by annual precipitation (AP, 58.3%) and the minimum temperature of the coldest month (MTCM, 24.3%), for a combined contribution of 82.6%. The response curves (**Figures 8d–f**) show that suitability increases markedly when AP ≥ 1, 900 mm and remains high when MTCM ≥ 2 °C. Although slope (SLP, 5.1%) contributes less overall, it can act as a local-scale modulator by influencing soil water retention and the near-surface microclimate, thereby structuring fine-grained patterns within suitable areas (Moore et al., 1991).

For *T. kirilowii*, the niche appears to be primarily climate-controlled, with MTCM (43.2%) and AP (25.9%) contributing most strongly, while elevation (ALT, 8.7%) and slope (SLP, 8.4%) reflect secondary topographic modulation at local scales. The response curves (**Figures 8g–i**) indicate a high-suitability range at AP of 500-1, 700 mm and higher suitability when MTCM lies between −4 and 4 °C. These results align with the view that microtopography shapes plant habitats by altering near-surface energy and moisture regimes (Scherrer and Körner, 2011).

### 3.4 Changes in potential suitable habitat under future climate scenarios

To investigate the future distributional dynamics of the three *Trichosanthes* species, multi-source environmental data were used to simulate the potential suitable habitats of *T. rubriflos*, *T. rosthornii*, and *T. kirilowii* with the MaxEnt model, and their distribution patterns were mapped under the present climate and four scenarios (SSP1-2.6, SSP2-4.5, SSP3-7.0, and SSP5-8.5) for 2060-2080.For easier comparison among species and scenarios, the main results on suitable habitat areas are summarized in **Table 3**.

**Table 3:** Suitable habitat areas for the three *Trichosanthes* species under different SSP scenarios (×10^4^ km²).

Species	Scenario	Area of suitable habitat (×10^4^ km²)			
		Expansion	Contraction	Stable	Net change
*Trichosanthes rubriflos*	SSP1-2.6	37.9	1.5	75.1	36.4
	SSP2-4.5	40.1	0.8	75.8	39.3
	SSP3-7.0	46.4	0.4	76.1	46
	SSP5-8.5	40.8	0.3	76.3	40.5
*Trichosanthes rosthornii*	SSP1-2.6	9.5	2.6	224.7	6.9
	SSP2-4.5	23.7	1.5	225.8	22.2
	SSP3-7.0	24.6	1.3	225.9	23.3
	SSP5-8.5	23.6	1.6	225.7	22
*Trichosanthes kirilowii*	SSP1-2.6	34	62.7	211.4	−28.7
	SSP2-4.5	37.2	43.5	230.6	−6.3
	SSP3-7.0	42.2	42.1	232	0.1
	SSP5-8.5	46.9	42.7	231.3	4.2

**Figure 9:**
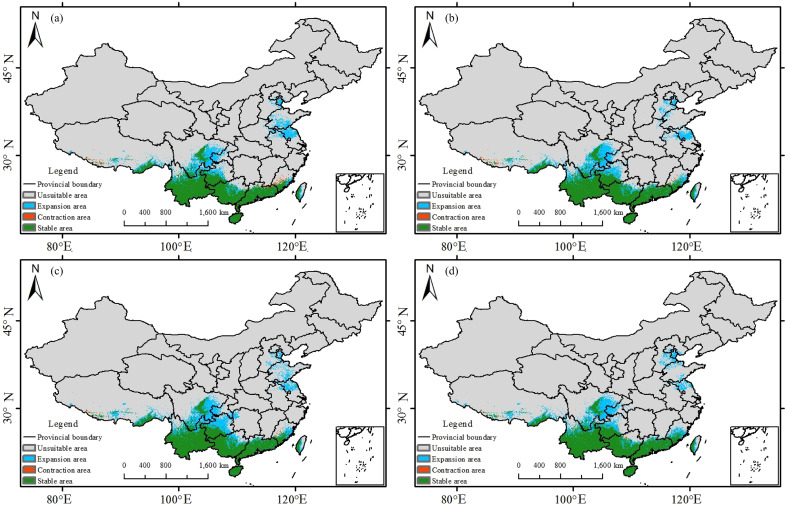
Potential suitable habitat of *Trichosanthes rubriflos* under the present climate and under 2060–2080 SSP scenarios: **(a)** SSP1-2.6; **(b)** SSP2-4.5; **(c)** SSP3-7.0; **(d)** SSP5-8.5.

**Figure 10:**
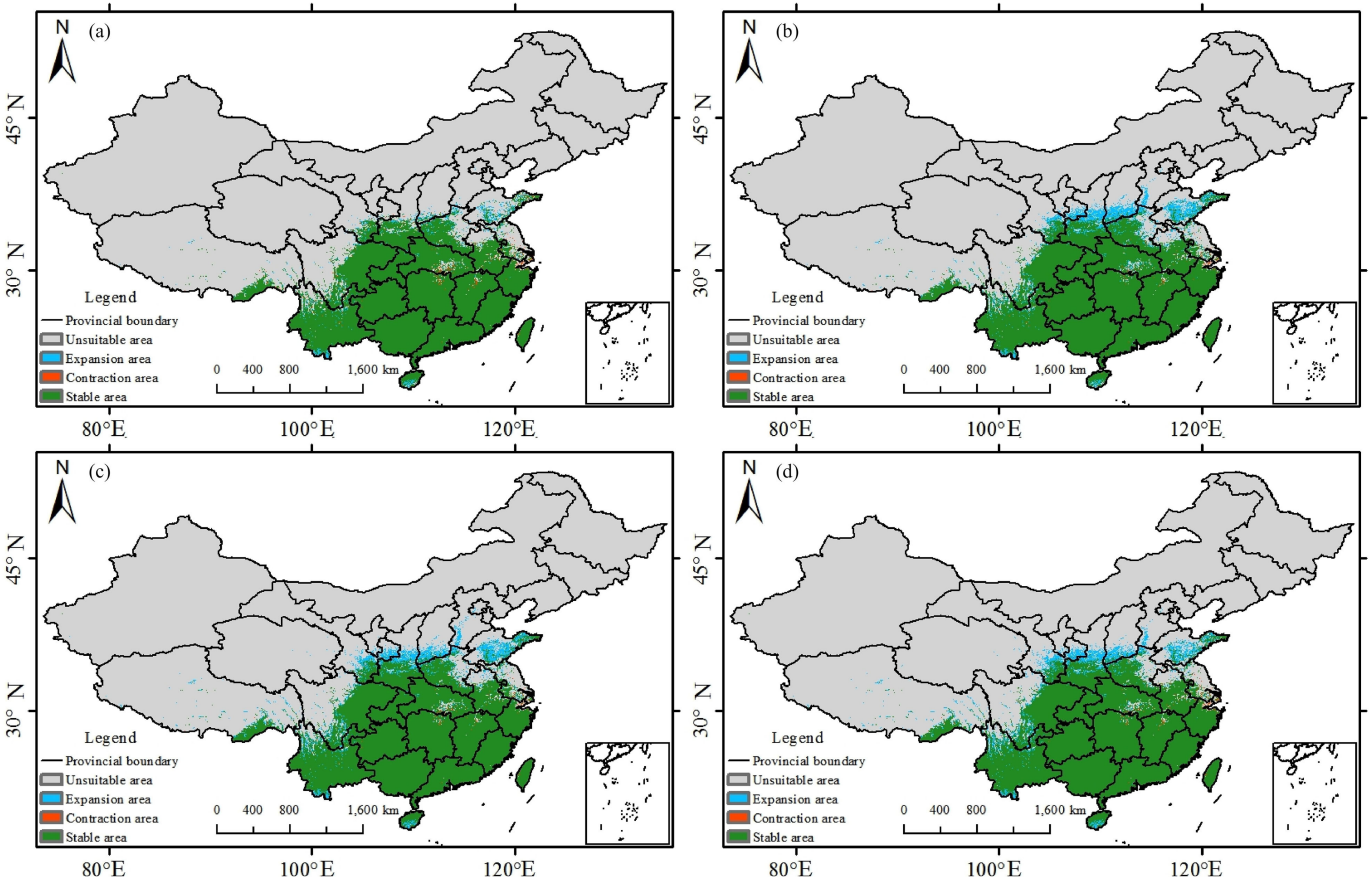
Potential suitable habitat distribution of *Trichosanthes rosthornii* under the current and 2060–2080 SSP scenarios: **(a)** SSP1-2.6; **(b)** SSP2-4.5; **(c)** SSP3-7.0; **(d)** SSP5-8.5.

**Figure 11:**
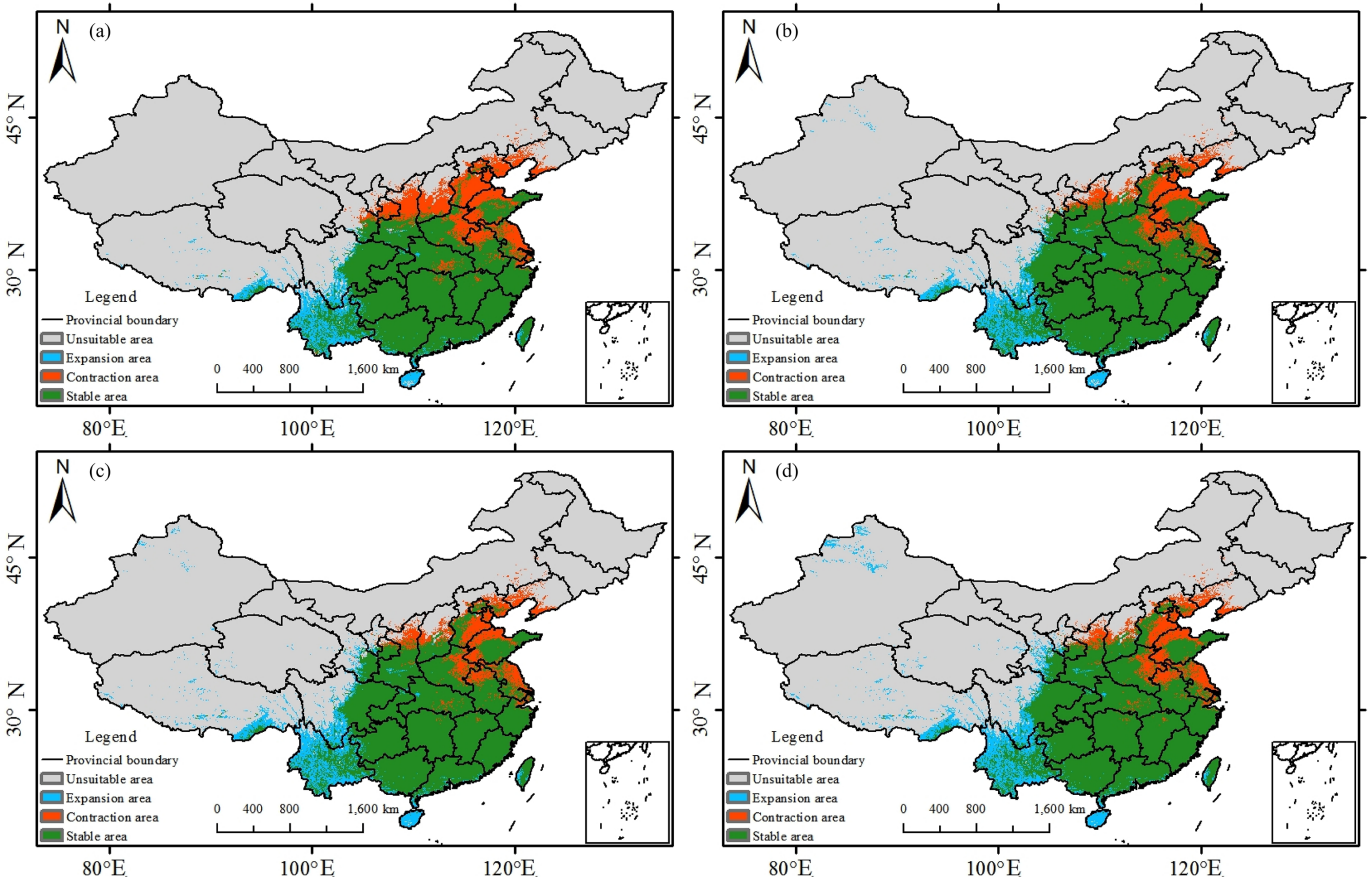
Potential suitable habitat distribution of *Trichosanthes kirilowii* under the current and 2060–2080 SSP scenarios: **(a)** SSP1-2.6; **(b)** SSP2-4.5; **(c)** SSP3-7.0; **(d)** SSP5-8.5.

Overall, the potential geographic ranges (**Figures 9**–**11**) of all three species tend to shift upslope and poleward under future climates. For *Trichosanthes rubriflos*, the distribution is predominantly stable across scenarios: the stable zone remains within 751, 000-763, 000 km². Although overall change is small, under SSP3-7.0 the expansion zone reaches 464, 000 km², while the contraction zone is minimal (30, 000 km²), indicating net expansion that outweighs contraction.

For *T. rosthornii*, stability is greatest, with the stable zone varying only slightly between 2, 247, 000 and 2, 259, 000 km². Expansion remains high under SSP2-4.5, SSP3-7.0, and SSP5-8.5 (236, 000-246, 000 km²) but is markedly lower under SSP1-2.6 (95, 000 km²). Contraction is consistently small (130, 000-260, 000 km²), suggesting that differences in emissions intensity have negligible influence on the core range and mainly drive limited edge expansion.

For *T. kirilowii*, expansion and contraction co-occur. The expansion zone increases from 340, 000 km² (SSP1-2.6) to 469, 000 km² (SSP5-8.5), indicating strong outward potential; however, contraction remains high (421, 000-627, 000 km²). Although the net change in area ranges from −287, 000 to +42, 000 km² (**Table 3**), the risk of persistent loss of core habitats remains evident, indicating substantially higher distributional uncertainty than for the other two species.

Overall, the potential geographic ranges of all three species tend to shift upslope and poleward under future climate scenarios.The interaction between temperature seasonality and annual precipitation strongly influences habitat suitability. Areas with moderate temperature fluctuations and sufficient annual precipitation provide the most suitable conditions for the three *Trichosanthes* species, whereas extreme values in either factor reduce habitat suitability. This interaction helps explain the spatial patterns of predicted distribution changes under different SSP scenarios.

## 4 Discussion

### 4.1 Key environmental determinants of the distributions of the three *Trichosanthes* species

For *T. rubriflos*, suitability is primarily driven by temperature seasonality (TS, 42.1%) and precipitation of the driest quarter (PDQ, 18.3%), which together account for 60.4% of the total contribution. The dominant role of TS indicates marked sensitivity to intra-annual thermal amplitude, with higher suitability under weaker temperature seasonality. The substantial PDQ effect points to dry-season water limitation as a key constraint. This accords with MaxEnt applications in which dry- and warm-season precipitation emerge as decisive drivers—for example, in distribution modelling of *Pinus dens flora* (Duan et al., 2022). Related work likewise emphasizes the importance of temperature seasonality and seasonal precipitation in delimiting suitable ranges of subtropical taxa (Wu et al., 2023).

For *T. rosthornii*, suitability is chiefly governed by annual precipitation (AP, 54.4%) and the minimum temperature of the coldest month (MTCM, 27.4%), for a combined contribution of 81.8%. The primacy of AP reflects strong dependence on water availability, whereas the high contribution of MTCM highlights cold stress as a major constraint on its distribution.

For *T. kirilowii*, response curves exhibit a double-threshold structure: suitability is high within intermediate ranges of temperature or precipitation but declines towards both lower and higher extremes. Such non-linear threshold effects have also been reported for medicinal plants in MaxEnt-based analyses (Yan et al., 2021).

### 4.2 Prediction of changes in the geographic distribution of *Trichosanthes* species under future scenarios

Across 2061–2080 under different SSP scenarios, overall, the observed expansions, contractions, and shifts of suitability zones closely correspond to projected changes in key climatic variables, such as temperature seasonality, annual precipitation, and minimum temperature of the coldest month. The three *Trichosanthes* species generally show range expansion, with *T. rubriflos* and *T. rosthornii* attaining their largest suitable areas under SSP3-7.0, whereas *T. kirilowii* peaks under SSP5-8.5. Further analyses were conducted to clarify patterns and mechanisms.

During 2061-2080, *T. rubriflos* remains broadly stable across the low-mountain and hilly belts of South and South-west China, expanding mainly along the northern Yunnan-Guizhou Plateau while showing minor local contraction in Fujian. AR6 notes that warming intensifies the global hydrological cycle with pronounced regional heterogeneity in precipitation (IPCC, 2021), and detection-attribution studies document a general weakening of temperature seasonality (TS) across China, including the south-west (Qian and Zhang, 2019). Under SSP3-7.0, reduced TS together with wetter conditions places the climate within the high-suitability range for *T. rubriflos*. Independent plateau-scale analyses likewise suggest that some taxa achieve larger high-suitability areas under intermediate forcing (e.g. SSP3-7.0) with partial retreat under higher forcing (e.g. SSP5-8.5), consistent with the localized contractions observed in this study (Dong et al., 2023).Overall, the observed expansions and contractions of *T. rubriflos* suitability zones closely correspond to projected changes in temperature seasonality and precipitation.

From 2061-2080, *T. rosthornii* is largely stable overall but shifts gradually northwards. Suitability concentrates in Shaanxi, Shanxi, and Ningxia, forming a narrow belt in western Shandong, while coastal zones and the middle-lower Yangtze exhibit localized contraction. Scenario comparisons indicate a peak suitable area under SSP3-7.0. Mechanistically, range frontiers of terrestrial plants are often constrained by low temperatures; progressive warming from SSP1-2.6 to SSP5-8.5 relaxes these cold limits, while precipitation responds heterogeneously (IPCC, 2021). Under SSP3-7.0, annual precipitation (AP) in the northern ecotone and along the eastern Loess Plateau more frequently falls within the species’ preferred interval, enhancing suitability and facilitating contiguous expansion; under SSP5-8.5, excessive warming and hydrothermal mismatch reduce suitability along the margins of the eastern lowlands, making SSP3-7.0 better aligned with the species’ hydrothermal requirements. The northward shift of *T. rosthornii*’s suitable range aligns with regional changes in annual precipitation and temperature patterns.

*T. kirilowii* shows a more pronounced redistribution: under SSP5-8.5, its suitable habitat contracts markedly in the north-eastern lowlands but expands persistently across the south-western mountains; the predicted reduction in suitability zones under SSP5-8.5 may partly reflect physiological constraints of the species under extreme warming (e.g., intolerance to higher temperatures or altered hydrothermal balance). However, such reductions may also arise from unresolved model limitations, such as the lack of fine-scale topographic refugia, unmodeled species interactions, and anthropogenic land-use effects, indicating that these projections should be interpreted cautiously.a continuous contraction belt emerges in Shandong, whereas pronounced expansion appears along the Sichuan Basin rim, the Yunnan-Guizhou Plateau, and the forelands of the Hengduan Mountains. Under SSP1-2.6 and SSP2-4.5, the magnitudes of warming and precipitation increase are insufficient to drive substantial south-westwards or upslope expansion; under SSP5-8.5, stronger warming and moisture changes, superimposed on the mountain-plateau topography of South-west China, promote elevational shifts. Over the past three decades, the equilibrium-line altitude (ELA) on Mount Gongga and in the western Himalaya has risen by 190–282 m (Guo et al., 2021), and dry-hot valleys at low elevations provide steep vertical gradients and topographic barriers, thereby facilitating movements towards higher elevations and more favorable microhabitats. As shown in **Figures 8g, h**, modest increases in AP and the minimum temperature of the coldest month (MTCM) benefit *T. kirilowii*, explaining its particularly strong expansion in the south-western mountains under SSP5-8.5.The redistribution of *T. kirilowii*’s suitable habitat is consistent with projected increases in minimum temperature of the coldest month and precipitation in the south-western mountains.

In summary, under SSP3-7.0, reduced TS and regional moistening bring hydrothermal conditions into the high-suitability range for *T. rubriflos*, yielding overall stability with outward expansion across the low-mountain and hilly belts of South and South-west China, alongside localized coastal contraction; in the northern ecotone and along the eastern margin of the Loess Plateau, AP falls within the preferred range of *T. rosthornii*, and under SSP3-7.0 its suitable range shifts slowly northwards; under SSP5-8.5, modest increases in AP and MTCM markedly raise suitability for *T. kirilowii* in the south-western mountains.

### 4.3 Uncertainty and implications of future habitat predictions

This study provides an integrated assessment of the current and future habitat suitability of three medicinal *Trichosanthes* species under climate change scenarios. In line with previous studies on *lianas*, our results reinforce the view that climbing plants play important roles in forest ecosystems by shaping vertical structure, light availability, and competitive interactions between canopy and understory vegetation (Schnitzer and Bongers, 2002; Ingwell et al., 2010; Schnitzer et al., 2014b). Accordingly, predicted shifts in suitable habitats of *T. rubriflos*, *T. rosthornii*, and *T. kirilowii* have implications not only for medicinal resource conservation but also for forest regeneration processes and ecosystem functioning.

The three species exhibited distinct responses to future climate scenarios, reflecting differences in climatic sensitivity and ecological strategies. Similar species-specific patterns have been reported in MaxEnt-based predictions for other medicinal plants, such as *Panax notoginseng* and *Glycyrrhiza* species, where suitability trajectories varied substantially among taxa under the same climate scenarios (Guo et al., 2024; Ma et al., 2025). These results suggest that conservation and management strategies should account for interspecific differences rather than relying on generalized approaches.

Despite the strong performance of the optimized MaxEnt models, several sources of uncertainty should be considered when interpreting future habitat predictions. Climate-related uncertainty primarily arises from differences among greenhouse gas emission pathways and climate model outputs, particularly in predictions of precipitation regimes and temperature extremes (IPCC, 2021). Such variability can propagate through species distribution models and affect both the magnitude and spatial patterns of predicted habitat shifts.

Model-related uncertainty is also inherent in species distribution modeling. Although MaxEnt is widely applied and performs well with presence-only data, predictions remain sensitive to parameterization choices, predictor selection, and the quality and spatial bias of occurrence records (Elith et al., 2011; Merow et al., 2013). While the tuning strategy adopted here reduced overfitting and improved model generalizability, these uncertainties cannot be fully eliminated.Furthermore, correlative models do not explicitly incorporate biotic interactions, dispersal limitations, or adaptive processes, which may lead to mismatches between predicted suitability and realized species distributions (Svenning et al., 2014; Park et al., 2022). For climbing plants such as *Trichosanthes*, dependence on host vegetation and forest structure may further constrain their responses to climate change, highlighting the need for caution when interpreting long-term predictions.

### 4.4 Limitations and outlook

Although this study employed a consistent set of climate drivers from the BCC-CSM2-MR model and used MaxEnt to project fine-scale future suitability, several limitations remain. These include limitations of the general circulation model in reproducing extreme and mesoscale climatic variability, the use of a single modeling algorithm which may not capture all ecological processes, and the exclusion of biotic interactions, land-use change, or microclimatic heterogeneity.First, general circulation models often struggle to reproduce extremes and mesoscale climatic variability, potentially understating or spatially misplacing threshold-type responses in species distributions. Second, the models here do not incorporate biotic interactions (e.g., competition, pollination), land-use change, or direct anthropogenic disturbance, thus simplifying the realized niche and warranting caution when translating projections into management decisions. Third, reliance on a single algorithm may introduce model selection bias. Future work should adopt ensemble species distribution modelling, integrate independent validation using field observations and remote sensing, and explicitly quantify uncertainty to improve robustness and accuracy.

The projected ranges of the three *Trichosanthes* species are shaped primarily by temperature seasonality (TS) and annual precipitation (AP), yet within-range migration pathways and microtopographic dependencies differ markedly. For multi-species conservation planning and sustainable cultivation, macroclimatic thresholds should be coupled with fine-scale filters—slope, aspect, and soil water-holding capacity—to support zone-based precision management that enhances the adaptive capacity and resource-use efficiency of these medicinal *Trichosanthes* vines under ongoing climate change.

## 5 Conclusions

MaxEnt was applied to integrate occurrence records for the three medicinal *Trichosanthes* species with 31 environmental variables, enabling estimation of factor contributions and sensitivities and projection of potentially suitable habitats under the present climate and for 2061 to 2080 across SSP1-2.6, SSP2-4.5, SSP3-7.0, and SSP5-8.5. The main conclusions are:

1. The optimized MaxEnt models showed excellent discrimination for all three species (all test-set AUCs ≥ 0.90), supporting the effectiveness of integrating the 31 environmental variables with occurrence data and confirming model reliability. Under the present climate, *T. rubriflos* forms a prominent and continuous core area over the Yunnan-Guizhou Plateau; *T. rosthornii* and *T. kirilowii* are more broadly distributed. T. *kirilowii* forms a continuous core belt across the hilly-plain zone of the middle-lower Yangtze, becoming progressively fragmented towards northern and south-western margins. Overall, the spatial patterns of *T. rosthornii* and *T. kirilowii* closely match the humid subtropical climate zone.
2. In terms of factor contributions, the potential distributions of the three species are dominated by climatic factors, whose aggregate contribution exceeds that of topography and soils. *T. rubriflos* is mainly driven by TS (42.1%) and PDQ (18.3%), with high suitability where TS is 125–630 and PDQ is 40–150 mm; *T. rosthornii* is dominated by AP (54.4%) and MTCM (27.4%), showing marked suitability where AP is ≥ 1, 900 mm and MTCM is ≥ 2 °C; *T. kirilowii* is chiefly driven by MTCM (43.2%) and AP (25.9%) and exhibits a double-threshold response, with high suitability where MTCM is −4 to 4 °C and AP is 500-1, 700 mm.
3. Across the four future scenarios, expansion predominates: expansion areas generally exceed contraction areas; at the genus-level there is a net northwards and upslope shift; and regional warming together with evolving precipitation patterns overall facilitates dispersal. *T. rubriflos* is largely stable. *T. rosthornii* remains highly stable, with only limited peripheral expansion. *T. kirilowii* tends to contract under SSP1-2.6 and SSP2-4.5 but expands under the other scenarios.
4. Under SSP3-7.0, reduced TS together with regional moistening brings hydrothermal conditions into the high-suitability range for *T. rubriflos*, yielding overall stability with outward expansion across the low-mountain and hilly belts of South and South-west China, alongside localized coastal contraction. Under the same scenario, AP in the northern ecotone and along the eastern margin of the Loess Plateau falls within the preferred interval for *T. rosthornii*, and its distribution shifts slowly northwards. By contrast, under SSP5-8.5, *T. kirilowii* is sensitive to modest increases in AP and MTCM, which markedly elevate suitability in the south-western mountains.

Future studies should focus on the long-term monitoring of these species’ distributions to track the changes predicted under different climate scenarios. Additionally, conservation measures could be based on these predictions, particularly for *T. kirilowii*, which may face contraction in certain regions under future climate scenarios. Identifying potential migration corridors and conserving key habitat areas in regions that are expected to be suitable for these species under future conditions could help mitigate the impacts of climate change.

## Data Availability

The datasets presented in this study can be found in online repositories. The names of the repository/repositories and accession number(s) can be found in the article/supplementary material.
