# Correction: MaxEnt model-based prediction of potential suitable habitats of three Trichosanthes L. species in China under future climate change scenarios

**DOI:** 10.3389/fpls.2026.1810021

**Published:** 2026-03-11

**Authors:** Xiaomeng Wang, Yuanyuan Ding, Chunfeng Duan, Yunchuan Xu, Chen Zhang, Zihan Wang

**Affiliations:** 1 Reading College, Nanjing University of Information Science and Technology, Nanjing, China; 2 School of Geographical Sciences, Nanjing University of Information Science and Technology, Nanjing, China; 3 Anhui Key Laboratory of Atmospheric Science and Satellite Remote Sensing, Anhui Institute of Meteorological Sciences, Hefei, China; 4 Taizhou Yuhuan Meteorological Bureau of Zhejiang, Taizhou, China; 5 Jiangsu Provincial University Key Laboratory of Agricultural and Ecological Meteorology, Nanjing University of Information Science and Technology, Nanjing, China

**Keywords:** climate change, MaxEnt, prediction, suitable habitat, Trichosanthes

There was a mistake in **Table 3** and its caption as published. The unit shown in parentheses was displayed as “(×10+ km²)”. The correct unit is “(×10^4^ km²)”. The corrected **Table 3**, and the corrected caption appears below.

**Table 3:** Suitable habitat areas for the three *Trichosanthes* species under different SSP scenarios (×10^4^ km²).

Species	Scenario	Area of suitable habitat (×10^4^ km²)			
		Expansion	Contraction	Stable	Net change
*Trichosanthes rubriflos*	SSP1-2.6	37.9	1.5	75.1	36.4
	SSP2-4.5	40.1	0.8	75.8	39.3
	SSP3-7.0	46.4	0.4	76.1	46
	SSP5-8.5	40.8	0.3	76.3	40.5
*Trichosanthes rosthornii*	SSP1-2.6	9.5	2.6	224.7	6.9
	SSP2-4.5	23.7	1.5	225.8	22.2
	SSP3-7.0	24.6	1.3	225.9	23.3
	SSP5-8.5	23.6	1.6	225.7	22
*Trichosanthes kirilowii*	SSP1-2.6	34	62.7	211.4	−28.7
	SSP2-4.5	37.2	43.5	230.6	−6.3
	SSP3-7.0	42.2	42.1	232	0.1
	SSP5-8.5	46.9	42.7	231.3	4.2

There was a mistake in **Figure 6** as published. Panels A-C of this figure were arranged in the wrong order. The sequence has now been corrected and the corrected **Figure 6** appears below.

**Figure 6:**
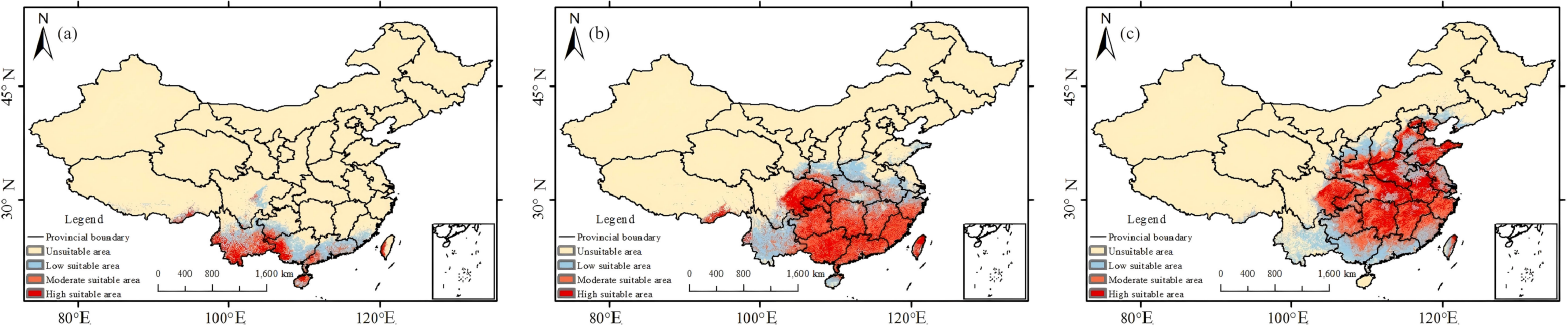
Present-day habitat suitability maps for the three *Trichosanthes* species: **(a)** *T. rubriflos*; **(b)** *T. rosthornii*, **(c)** *T. kirilowii*.

The images for **Figures 9** and **10** were erroneously swapped in the published article. The correct version of **Figures 9** and **10** appear below.

**Figure 9:**
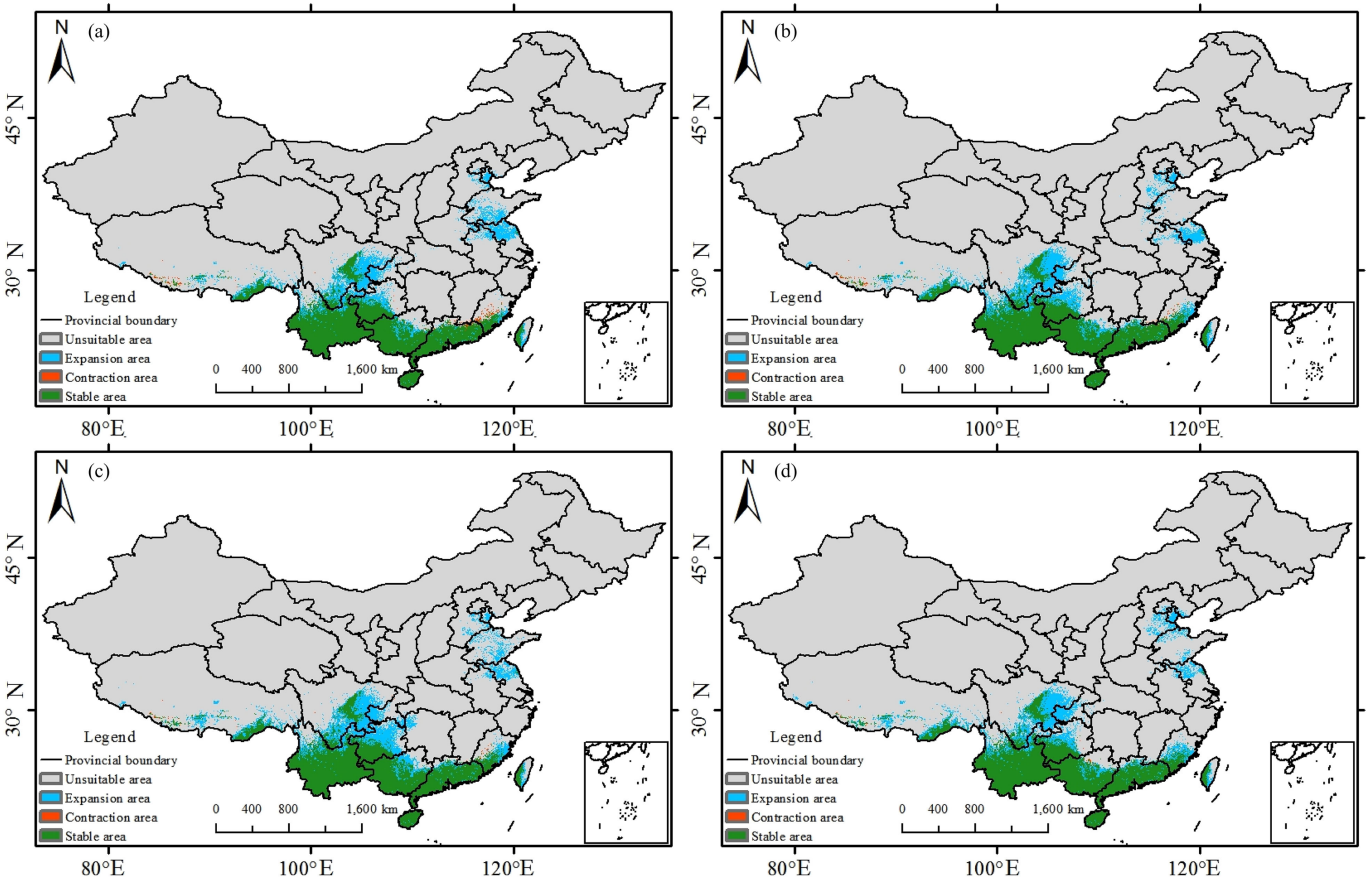
Potential suitable habitat of *Trichosanthes rubriflos* under the present climate and under 2060–2080 SSP scenarios: **(a)** SSP1-2.6; **(b)** SSP2-4.5; **(c)** SSP3-7.0; **(d)** SSP5-8.5.

**Figure 10:**
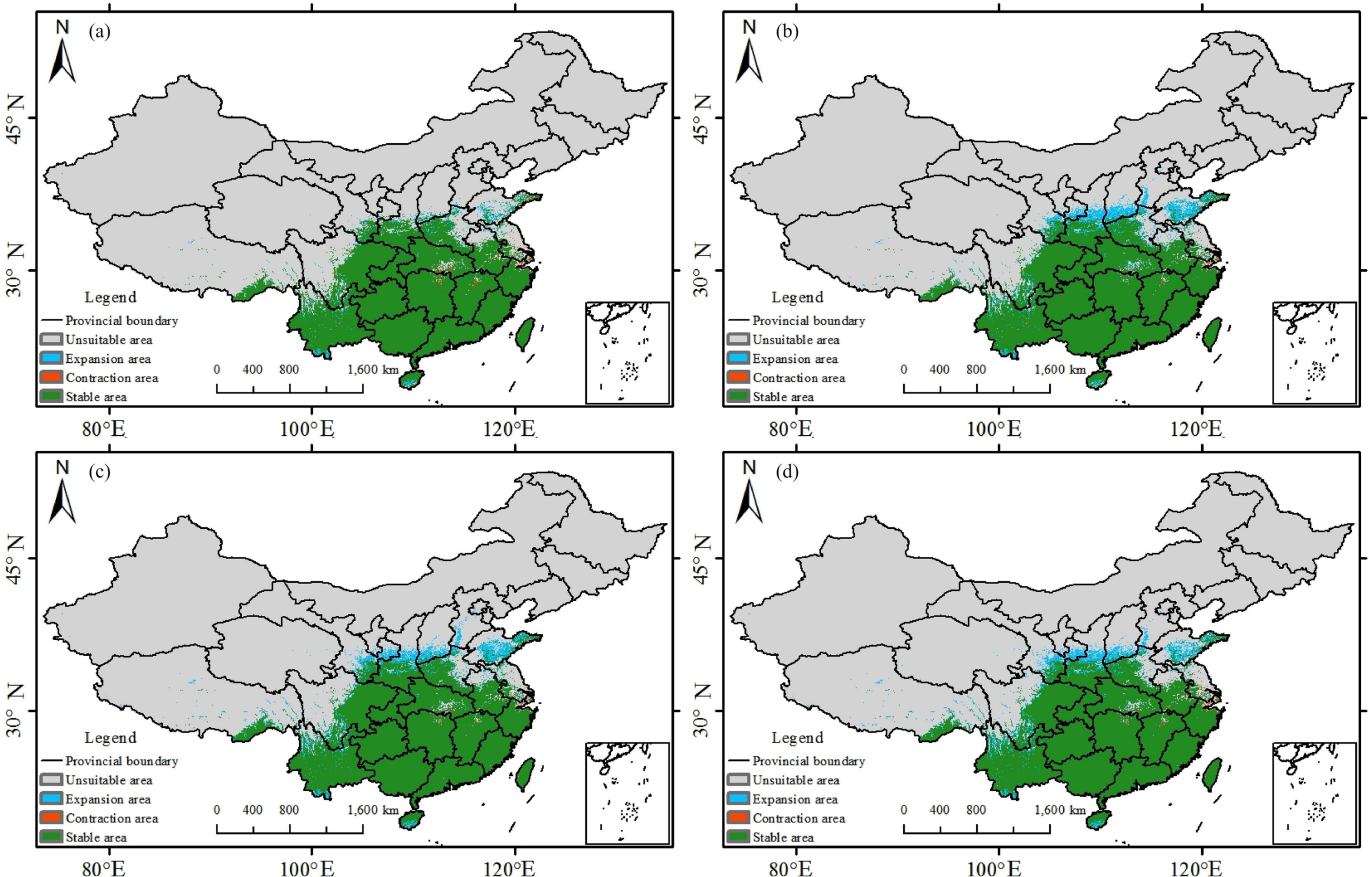
Potential suitable habitat distribution of *Trichosanthes rosthornii* under the current and 2060–2080 SSP scenarios: **(a)** SSP1-2.6; **(b)** SSP2-4.5; **(c)** SSP3-7.0; **(d)** SSP5-8.5.

The original version of this article has been updated.

